# Magnetic Resonance Imaging Evaluation of Closed-Mouth TMJ Disc-Condyle Relationship in a Population of Patients Seeking for Temporomandibular Disorders Advice

**DOI:** 10.1155/2021/5565747

**Published:** 2021-12-02

**Authors:** Matteo Tresoldi, Ricardo Dias, Alessandro Bracci, Marzia Segù, Luca Guarda-Nardini, Daniele Manfredini

**Affiliations:** ^1^Private Practice in Dentistry, Milan, Italy; ^2^Institute of Oral Implantology and Prosthodontics, Dentistry Department, Faculty of Medicine, University of Coimbra, Coimbra, Portugal; ^3^Neuroscience Department, University of Padova, Padua, Italy; ^4^Department of Clinical-Surgical, Diagnostic and Paediatric Sciences, University of Pavia, Pavia, Italy; ^5^Section of Dentistry and Maxillofacial Surgery, Treviso Hospital, Treviso, Italy; ^6^School of Dentistry, University of Siena, Siena, Italy

## Abstract

**Objective:**

To characterize the closed-mouth temporomandibular joint (TMJ) disc-condyle relationship in a population of individuals who sought hospital services for temporomandibular disorders (TMD).

**Methods:**

Two hundred and twenty-four TMJ magnetic resonance images (MRIs) of 112 patients were assessed in all spatial planes to classify disc position with respect to the condyle in a closed-mouth position.

**Results:**

Disc displacement (DD) was present in 62.1% and superior disc position in 29.9% of the patients. Position could not be determined in 8% of the cases. Among DD, pure anteriorized position was the most common condition (34.4%), with different combined translational and rotational displacements in all the other joints (27.7%).

**Conclusion:**

There is a wide biological variability in disc position in closed mouth among patients seeking for TMD advice. Getting deeper into the correlation with clinical symptoms is recommended to refine the potential relevance of any diagnostic and management strategies based on the imaging evaluation of TMJ disc position.

## 1. Introduction

Temporomandibular disorders (TMDs) are a group of musculoskeletal pathologies involving the temporomandibular joint, the jaw muscles, and related structures [1]. The etiology is multifactorial, with multiple interacting local and systemic risk factors [2–4]. Muscle and/or temporomandibular joint (TMJ) pain, joint sounds, and movement limitations are the most frequent signs and symptoms, which make TMDs the most common cause of nonodontogenic orofacial pain [5]. They are sometimes associated with other symptoms and comorbid conditions, such as headache, ear-related symptoms, and neck dysfunction [6]. TMD prevalence is around 5–12% of the adult population, occurring more frequently in females aged between 20 and 40 years [7, 8]. TMDs can be classified into articular and/or muscle disorders [9]. Among the former, degenerative joint disorders and disc displacements are the most commonly studied conditions [10].

The TMJ is a bilateral, complex, and critical load-bearing joint. The articular disc acts as a stress absorber, and its positional relationship with the TMJ condyle has been much studied. In particular, several investigations described the positional changes on the sagittal plane with respect to the purported reference position [11]. In parallel, many speculations have been made with regard to the need for an ideal position as a target for warranting good function and absence of symptoms.

For the evaluation of TMJ soft tissues, magnetic resonance imaging (MRI) is the reference technique [12–15]. Such a technique combines the advantage of allowing the observation of soft tissues as well as the possible presence of intra-articular fluid accumulation in nearly any desired plane of reference [16]. The diagnostic accuracy of MRI for the assessment of the TMJ disc position and morphology is 95% with respect to autopsy specimens [17]. Currently, MRI is considered a requirement for second-step evaluations of internal derangements and it is recommended for the implementation of clinical assessment by the reference academy guidelines [18].

Disc-condyle relationships other than the so-called superior position (i.e., with the posterior band of the disc located at around 12 o'clock with respect to the condylar head) have historically been considered abnormal. A disc displacement (DD) may be due to factors intrinsic to the TMJ itself (e.g., anatomical predisposition) or to external factors (e.g., forces applied to the mandibular condyle that influence the relationship of joint components and/or determine tissue changes) [19]. On the other hand, emerging knowledge suggests that a wide range of positional biological variations may exist for disc position within the temporomandibular joint, not only in healthy individuals but also in TMD patients. This means that getting deeper into this issue by a characterization of the full spectrum of possible disc-condyle relationships could clarify the clinical relevance of studying the disc-condyle relationship and the implications of MRI for diagnosing and planning the management of TMJ disorders [11, 20].

Within these premises, this study aims to characterize the closed-mouth disc-condyle relation in all spatial planes (i.e., sagittal, frontal, and coronal) and all possible positions in a nonselected population of individuals who sought hospital services for TMD.

## 2. Materials and Methods

This is an observational study of the MRIs of 112 patients (91 females; mean age 52 yo) attending the Treviso Hospital, Italy, between January and May 2019. Patients asked for advice because of TMD signs and symptoms. In this study, the MRIs of patients that satisfied any diagnostic criteria for temporomandibular disorders (DC/TMD) for joint disorders and that reported the signs/symptoms for more than three months were included [9]. MRIs were performed as a complementary diagnostic test. All patients underwent bilateral TMJ MRI to assess disc position with respect to the condyle in a closed-mouth position. The Institutional Review Board of Treviso Hospital (Italy) provided approval for this observational study, in accordance with the Declaration of Helsinki (1964).

All MRIs were collected in the same center and by the same radiologist with expertise in TMJ imaging interpretation, using a MAGNETOM Avanto-Fit 1.5 T scanner (Siemens Healthcare GmbH, Germany). The subjects were positioned in the supine position, with the sagittal plane perpendicular to the horizontal plane and the Frankfurt plane parallel to the scanner gantry. Oblique parasagittal slices were obtained and corrected by the horizontal angulation of the condyle in a closed-mouth position. A 2 mm slice thickness in sequential sagittal, coronal, and frontal T1-weighted images was obtained in the closed-mouth position, with a 140 mm field of view and spin-echo multisection images (repetition time and echo time were 510–520 ms and 11–15 ms, respectively). A single observer assessed the images in order to classify the disc position in closed mouth according to the classification of Tasaki et al. [13].

**Figure 1:**
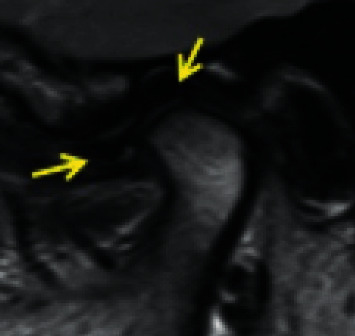
Temporomandibular joint superior disc position.

*Superior disc position*: the posterior band of the disc is superior to the condyle, or the central thin zone of the disc is located between the anterior prominence of the condyle and the posterior aspect of the articular eminence (Figure 1).

**Figure 2:**
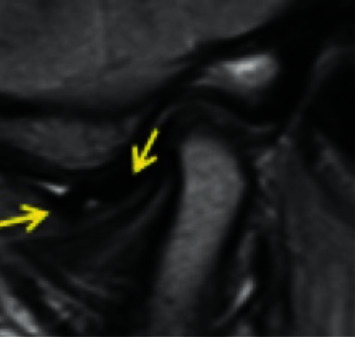
Temporomandibular joint anterior disc displacement.

*Anterior disc displacement*: the posterior band of the disc is anterior to the anterior condylar prominence throughout the mediolateral dimension of the joint (no rotational or mediolateral component of disc displacement) (Figure 2).

**Figure 3:**
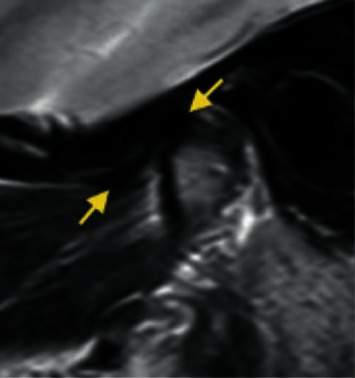
Temporomandibular joint partial anterolateral disc displacement.

*Partial anterior disc displacement in the lateral part of the joint*: the disc is anteriorly displaced in the lateral part of the joint and is in a superior position in the medial part of the joint with no sideways component of displacement (Figure 3).

**Figure 4:**
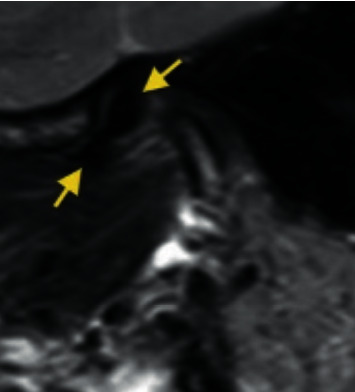
Temporomandibular joint partial anteromedial disc displacement.

*Partial anterior disc displacement in the medial part of the joint*: the disc is anteriorly displaced in the medial part of the joint and is in a superior position in the lateral part of the joint with no sideways component of displacement (Figure 4).

**Figure 5:**
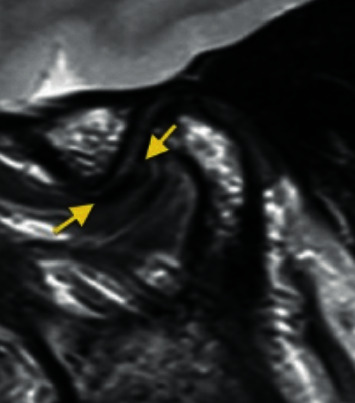
Temporomandibular joint rotational anterolateral disc displacement.

*Rotational anterolateral disc displacement*: the disc is anteriorly and laterally displaced (Figure 5).

**Figure 6:**
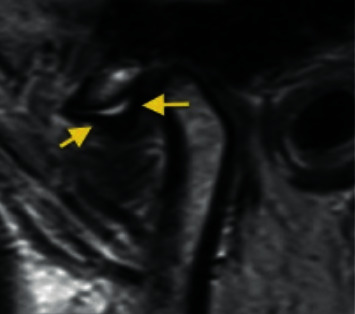
Temporomandibular joint rotational anteromedial disc displacement.

*Rotational anteromedial disc displacement*: the disc is anteriorly and medially displaced (Figure 6).

**Figure 7:**
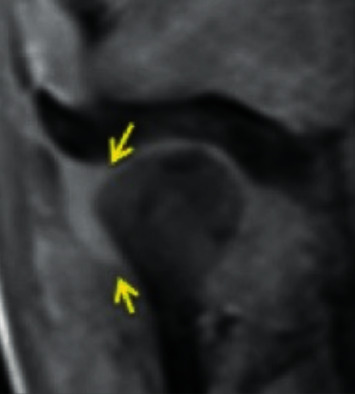
Temporomandibular joint lateral disc displacement.

*Lateral disc displacement*: the disc is displaced laterally to the lateral condylar pole (Figure 7).

**Figure 8:**
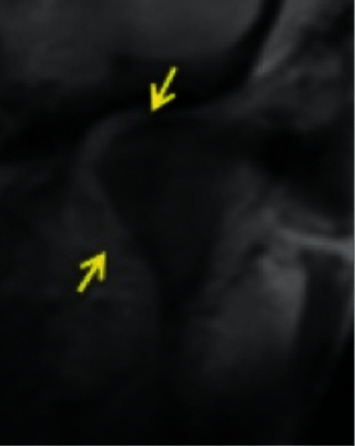
Temporomandibular joint medial disc displacement.

*Medial disc displacement*: the disc is displaced medially to the medial condylar pole (Figure 8).

**Figure 9:**
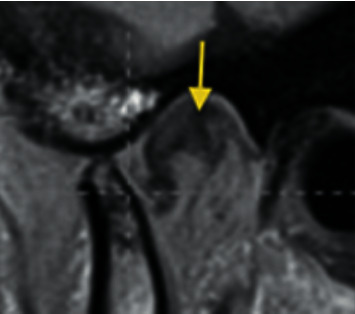
Temporomandibular joint posterior disc displacement.

*Posterior disc displacement*: the disc is displaced posteriorly to the 12 o'clock position on top of the condyle (Figure 9).

**Figure 10:**
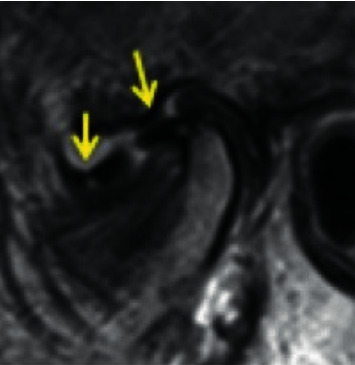
Temporomandibular joint indeterminate disc position.

*Indeterminate*: this category was used when a large perforation, prior surgical therapy, or no clear image of the disc prevented classification into any of the abovementioned categories (Figure 10).

A descriptive analysis of the percentage frequency for each disc position was performed. For descriptive purposes, each TMJ was considered as a unit.

## 3. Results

Two hundred and twenty-four TMJ images were assessed of 112 patients (91 females; mean age 52 yo). The characterization of the sample by sex, age, and orofacial pain score according to the visual analogue scale (VAS) is presented in Table 1. The frequency of pain and/or joint disorders according to DC/TMD is presented in Table 2. Most patients presented with disc displacement (62.1%), whilst 29.9% had a superior disc position. Within the joints with disc displacement, anterior disc position (Figure 2) was the most frequent condition (34.4%), but a wide variability in the direction and rotation of displacement is shown in Table 3.

The disc position could not be determined in 8% of the joints, and a posterior disc displacement was not found in any of the TMJ.

## 4. Discussion

This investigation described the TMJ disc position in a population of patients seeking for TMD advice. The diagnostic characterization of the sample demonstrates the variability found when open classification systems like DC/TMD are used, which allow the inclusion of a patient in multiple categories. Furthermore, it demonstrates the heterogeneity of clinical conditions that translate into pain in the orofacial area lasting more than 3 months and are often simply categorized as TMDs, in line with literature using similar methodology [21]. Care must be taken when reading and interpreting a population of TMD patients. Although they all look the same, they may be representative of different population subgroups.

The purpose of this study was to provide a standpoint for future comparisons based on a patient sample of individuals that may be representative of a TMJ disorder population. MRI is considered the reference imaging technique for TMJ conditions since it allows the simultaneous evaluation of the morphology and position of the articular disc and bone structures of the TMJ, in addition to evaluating the functional relationships between the condyle, articular disc, mandibular fossa, and articular eminence. A multisection analysis of MRI images allows distinguishing the normal disc position from disc displacement and can improve the possibility to distinguish between various stages of intra-articular derangement of TMJ. For optimal imaging of the TMJ, small bilateral surface coils with a small field of view were used to achieve a higher signal-to-noise ratio and simultaneous bilateral acquisition. The closed-mouth coronal and frontal T1 sequences were used to evaluate the overall anatomy, disc position, and intra-articular arrangement. Only the closed-mouth position was considered in our study since it is in this position that the condyle/disc relationship is categorized by the Tasaki et al. classification [13]. The open-mouth position determines if there is a change/modification of this relationship with the opening movement, which was beyond the scope of our study. This is an observational study, which merely analyzed the MRI of patients who sought for advice in the hospital and who underwent MRI to complement the TMD clinical diagnosis. The design of a case-control study with asymptomatic individuals might have been useful to add information on the topic, but obvious practical and ethical concerns prevented its realization. The inclusion of a control group of individuals who underwent MRIs for other reasons or investigations of other head areas might add interesting information, but unfortunately, MRIs not focusing on the TMJ do not have the characteristics usually required for an adequate TMJ evaluation.

To the best of our knowledge, this investigation is one of the first to depict the full spectrum of disc locations with respect to the condyle, thus adding information to the literature studies describing the disc-condyle relation only on the sagittal or coronal planes. Based on that, the findings may offer some interesting points for discussion.

As a general remark, findings are in accordance with the literature, showing a high frequency of disc displacement in TMD populations, which is generally higher than in healthy individuals. Tasaki et al. evaluated 600 MR images of 243 TMD patients and 57 volunteers and found disc displacements of different stages in the sagittal plane in 80% of the patients as compared with 30% of the volunteers. A successive study by Larheim and Westesson (2001) also showed a higher frequency of complete anterior and anterolateral disc displacements in patients with TMD than in healthy controls [22].

This study confirmed that the frequency of MRI-depicted disc displacement is high among TMD patients. The so-called physiological superior disc position was found in 29.9% of joints, an undetermined position was shown in 8%, whilst a displacement of a different type and direction was depicted in all the other joints. Amongst those, an anterior position is the most frequent condition (34.4%). These values are in agreement with the study of Emshoff et al. (2002), who reported 64.9% of disc displacements in patients with TMJ pain, of which 34.9% were anterior displacements [11]. Posterior displacements were not identified in the current study in any TMJs, which is also in accordance with the rare frequency described in the literature [23, 24]. For instance, De Farias et al. (2015) found 1.1% of joints with posterior displacement [25].

Whilst these findings are supportive of disc-condyle incoordination in the majority of TMD patients, it should nonetheless be noted that even individuals with a “normal” (i.e., superior) disc position might require TMD advice. In particular, a little more than one out of four joints did not have an abnormal disc position. The diagnostic variability of our sample also reinforces this need for further studies and understanding of diagnosis and treatment decision trees. This reflects the need for adequate clinical exploration and for an imaging prescription as a complement to clarify certain clinical conditions. This also suggests that a careful evaluation of the correlation between imaging and clinical studies is recommended before assuming that evaluation of disc position is a fundamental factor to explain the clinical picture. Early findings on symptom-free populations showing the presence of disc displacement may support this cautionary statement [26].

Altogether, these findings are open to several considerations.

First, the fact that an anterior displacement is the most frequent condition can be related with the anatomical peculiarity of the disc. Its biconcave shape, with a thinner posterior than anterior band, may predispose to a natural tendency to “slide” anteriorly. Besides, the anterior and collateral ligaments may tend to “trap” the disc to a more anterior position. The different elastic characteristics of the ligaments, associated with the nonsymmetrical and nonlinear loads over the TMJ between the medial and lateral sides of the joint, could explain why the third most frequent disc position was the partial anterolateral displacement [27]. A partial anterior disc displacement in the lateral part of the joint was identified in 13% of the joints, in accordance with the 11% observed by Foucart et al. (1998) [24]. The presence of an intra-articular adaptive process and/or issues related with the complex differential diagnosis of orofacial pain may explain the superior disc position that was observed in 23% of the joints in the present study. Interestingly, this finding is in agreement with the 26% and 29.5% reported by Foucart et al. (1998) and De Farias et al. (2015), respectively [24, 25].

Second, a wide biological variability in the disc-condyle relationship exists in the different planes. Based on that, it is recommended that future work seeks to assess the correlation between specific disc displacements and clinical signs and symptoms, given the noisy annoyance represented by click sounds [28]. The working hypothesis might be that specific clusters of signs and symptoms are indicative of a certain disc-condyle relationship. On the other hand, it can be speculated that such a direct relationship is unlikely to be found due to the number of different disc positions and the complexity of possible clinical pictures. This information is nonetheless important to alert against any disc repositioning approaches for symptom-relief purposes. Restorative plans based on a change or redefinition of intra-articular positional relationships cannot be a warranty of stability or positional maintenance over time. Furthermore, integrated analysis of disc position evaluation in the open mouth can be added to future studies.

Third, the possible association between disc displacement and some condylar shapes might be considered for future investigations. Some studies found a higher prevalence of anterior disc displacement in convex and rounded condyles, as well as in angled condyles [29]. Another study evaluated condyle dimensions and found an association of disc displacement with a narrow condylar size in the anteroposterior and transverse directions [30]. Inconsistency in findings may be associated with different methodologies that have been adopted for evaluating condylar morphology and disc displacement. On the other hand, the biconcave shape of the TMJ disc is considered the physiological macrostructure [25]. Morphological changes of the TMJ condyle (e.g., degenerative processes and fractures) and/or of the disc itself (perforated disc and broken disc) seem to be relevant conditions in jeopardizing the positional stability of the disc.

Fourth, the fact that some patients did not present any imaging abnormalities suggests that images could not be used as a stand-alone diagnostic finding. In 23% of the study joints, the disc-condyle relationship was physiological, as interpreted based on current diagnostic guidelines, and can be taken as an example of the possible usefulness of MRI to exclude intra-articular disorders [31]. A diagnostic and therapeutic approach based only on MRI evaluation can result in under- or overtreatment.

## 5. Conclusion

There is a relevant frequency and variability of combined positional and rotational MRI displacements of the TMJ disc among patients seeking TMD advice. Based on MRIs, about 30% of joints have a physiological disc-condyle relationship. Based on these findings, it is hardly arguable that MRI disc position may have a clear-cut correlation with clinical signs and symptoms. Thus, while it is recommendable that symptom management strategies are not orientated by the position of the disc or by imaging signs, there is surely a need to understand how much of the biological variability of the disc-condyle relationship is actually part of a natural course of joint wear and ageing and how much of it is the result of joint overload.

## Figures and Tables

**Table 1:** Characterization of the sample by sex, age, and orofacial pain score according to the visual analogue scale (VAS).

Sex	Age range	n	Age (mean)	Orofacial pain (VAS score: mean)
Male	17–39	22	28	5
	40–59	33	48	5
	>60	36	72	5
	Total	91	50	5

Female	17–39	8	26	4
	40–59	6	52	4
	>60	7	73	5
	Total	21	50	4

**Table 2:** Frequency of TMD diagnosis according to DC/TMD in 112 patients. Some of the patients presented more than one diagnosis.

Diagnostic according to DC/TMD	Cases	Frequency
*Pain disorders*		
Local myalgia	9	8
Myofascial pain	0	0
Myofascial pain with referral	0	0
Arthralgia	93	83.2
Headache attributed to TMD	0	0

*Joint disorders*		
Disc displacement with reduction	13	11.6
Disc displacement with reduction and intermittent locking	0	0
Disc displacement without reduction and with limited opening	4	3.6
Disc displacement without reduction and without limited opening	0	0
Degenerative joint disease	2	1.6
Subluxation	0	0

**Table 3:** TMJ disc-condyle relationship in closed mouth (*N*  = 224 joints).

Disc position and displacement	Cases	Frequency
Superior disc position	67	29.9
Anterior disc displacement	77	34.4
Partial anterior disc displacement in lateral part of joint	28	12.5
Partial anterior disc displacement in medial part of joint	1	0.4
Rotational anterolateral disc displacement	8	3.6
Rotational anteromedial disc displacement	1	0.4
Medial disc position	14	6.3
Lateral disc position	10	4.5
Posterior disc position	0	0
Indeterminate	18	8
Total	224	100%

## Data Availability

The data used to support the findings of this study are available from the corresponding author upon request.
